# Efficacy and Safety of 5 µg and 10 µg Dexmedetomidine With Hyperbaric Levobupivacaine in Lower-Limb Surgeries Under Spinal Anesthesia: A Randomized Controlled Study

**DOI:** 10.7759/cureus.97805

**Published:** 2025-11-25

**Authors:** Anmol Bhujabal, Laxman Kumar Senapati, Pinky Garg, Ganesh C Satapathy

**Affiliations:** 1 Anesthesiology, Kalinga Institute of Medical Sciences, KIIT Deemed to Be University, Bhubaneswar, IND; 2 Anesthesiology, United Institute of Medical Sciences, Prayagraj, IND

**Keywords:** dexmedetomidine, hemodynamic stability, levobupivacaine, lower-limb surgery, spinal anesthesia

## Abstract

Background: Dexmedetomidine is used as an intrathecal adjuvant to enhance the quality and duration of spinal anesthesia (SA). However, the extent to which different intrathecal doses affect the characteristics of the block and hemodynamic responses remains unclear.

Objective: To compare the efficacy and safety of intrathecal 5 µg versus 10 µg dexmedetomidine added to 0.5% hyperbaric levobupivacaine in patients undergoing lower-limb surgery.

Methods: 60 American Society of Anesthesiologists (ASA) I-II adults scheduled for elective lower-limb orthopedic and soft-tissue procedures were randomized into two groups. Group D5 received 5 µg dexmedetomidine, and Group D10 received 10 µg intrathecally with hyperbaric levobupivacaine. Sensory and motor block characteristics were assessed every three minutes until maximum levels were achieved. Hemodynamic parameters, including heart rate (HR), systolic blood pressure (SBP), diastolic blood pressure (DBP), and mean arterial pressure (MAP), were monitored at predefined intervals. The primary outcome was the duration of sensory block; secondary outcomes included onset time, motor block duration, and intraoperative hemodynamic changes.

Results: Group D10 reached maximum sensory block (3.65 ± 1.15 vs. 4.40 ± 1.22 min; p = 0.017) and maximum motor block (6.05 ± 1.30 vs. 7.90 ± 2.12 min; p = 0.0001) significantly faster than Group D5. The duration of sensory block was also longer in Group D10. Bradycardia and hypotension occurred more frequently in Group D10 but were transient and managed without complications.

Conclusion: Both doses enhanced SA characteristics. The 10 µg dose produced faster onset and longer duration of block but with a higher incidence of manageable hemodynamic effects. Selection of dose should balance the need for prolonged anesthesia with safety considerations.

## Introduction

According to the revised International Association for the Study of Pain (IASP) Task Force 2018-20, pain is defined as an unpleasant sensory and emotional experience associated with, or resembling that associated with, actual or potential tissue damage [1]. It is a subjective experience shaped by a combination of psychological, biological, and social factors. Control of pain is an essential component of perioperative care, with a significant impact on the outcome of surgery and the patient's recovery. Ideal intraoperative pain control reduces the physiological stress response, enables earlier mobilization, and decreases postoperative morbidity. Anesthesiologists are thus continually seeking the best anesthetic methods that provide enhanced analgesia with a reduced side-effect profile [2].

In lower-limb surgeries, spinal anesthesia (SA) is favored over alternative anesthetic modalities owing to its procedural simplicity, cost-benefit profile, and ability to provide quicker onset of both motor and sensory block [3]. Of the local anesthetics used, bupivacaine is extensively used. It is the racemic mixture of its enantiomers, dextrobupivacaine, and levobupivacaine. The pure S-optical isomer, levobupivacaine, is becoming increasingly popular as a safer alternative due to its reduced cardiotoxicity and central nervous system depression. It possesses a favorable pharmacokinetic and pharmacodynamic profile and is therefore an option for regional anesthesia (RA) [4]. Nevertheless, its duration of action may not suffice for moderately long orthopedic procedures, such as open reduction and internal fixation, tibial plating, or femoral nailing, which typically last 90-150 minutes. Therefore, an effective and safe adjuvant is essential to extend the duration of SA without increasing postoperative complications.

Recently, adjuvants for RA have been increasingly used [5]. α2-adrenergic receptor agonists have been used as useful adjuvants to RA and general anesthesia (GA) due to their sympatholytic effects. These drugs exert their effects by stimulating prejunctional inhibitory α2-adrenoceptors, which inhibit the release of norepinephrine. Inhibition of signal conduction by presynaptic and postsynaptic mechanisms results in anxiolysis, analgesia, and sedation [6]. Dexmedetomidine, a member of this drug class, has demonstrated exceptional potential as a complement to RA by augmenting the effects of local anesthetics and extending analgesia [7]. Previous studies have explored intrathecal doses ranging from 3 µg to 10 µg, demonstrating improved analgesic efficacy but a dose-dependent risk of bradycardia and hypotension due to sympatholytic effects [7].

Despite this growing evidence, there is no consensus on the optimal intrathecal dose that achieves the desired anesthetic prolongation while maintaining hemodynamic safety. Doses at the lower end (3-5 µg) are generally well tolerated but may offer limited duration, whereas higher doses (8-10 µg) produce longer anesthesia with greater risk of cardiovascular effects [7]. Intermediate intrathecal dexmedetomidine doses (6-9 µg) have been assessed in previous studies, but the results consistently show that these doses do not provide clinically superior prolongation of sensory or motor blocks compared with the widely studied 5 µg dose. For example, Saha et al. and Halder et al. demonstrated that doses of 7-8 µg produced block durations comparable to 5 µg while still exhibiting the dose-dependent bradycardia and hypotension typically seen with higher doses [8,9]. Because intermediate doses fail to offer additional clinical benefit yet retain the hemodynamic risks of the higher dose range, the present study focused on comparing the most commonly recommended minimum effective dose (5 µg) with the upper acceptable dose (10 µg) to establish a clear efficacy-safety threshold

In this context, we designed a randomized controlled study to compare the efficacy and safety of 5 µg versus 10 µg of intrathecal dexmedetomidine when added to 0.5% hyperbaric levobupivacaine in patients undergoing elective lower-limb surgeries. By evaluating onset time, block duration, and hemodynamic changes, this study aimed to identify the dose that provides an optimal balance between prolonged anesthesia and hemodynamic stability, thereby addressing a clinically relevant gap in anesthetic dosing strategies for SA.

This study hypothesizes that varying doses of dexmedetomidine, when combined with hyperbaric levobupivacaine, differ in their efficacy and safety profiles in prolonging the duration of sensory block in lower-limb surgeries under SA. The primary objective of this study was to compare the duration of sensory block between intrathecal dexmedetomidine doses of 5 µg and 10 µg when combined with hyperbaric levobupivacaine. The secondary objectives were to compare the onset times of sensory and motor block, the duration of motor block, the time to regression to T8, and the intraoperative hemodynamic changes and adverse event profiles between the two groups. These defined objectives were designed to determine the optimal dose-balancing efficacy and safety.

## Materials and methods

Study design, ethical consideration, and trial registration

This randomized, double-blind, interventional trial was conducted in the Department of Anesthesiology and Critical Care at the Kalinga Institute of Medical Sciences, Pradyumna Bal Memorial Hospital, Kalinga Institute of Industrial Technology, Deemed to Be University, Bhubaneswar, India, after obtaining written informed consent from all participants. The study period extended from December 2023 to February 2025. Ethical approval was obtained from the Institutional Ethics Committee (Approval No. KIIT/KIMS/IEC/1211/2023), and the trial was registered prospectively with the Clinical Trials Registry of India (CTRI/2023/11/060207, dated November 23, 2023).

Eligibility criteria

The study included individuals aged 18 to 60 years of the American Society of Anesthesiologists (ASA) physical status I or II scheduled for lower-limb surgical procedures [10]. Participants were excluded if they met any of the following criteria: a body mass index greater than 30 kg/m², a history of adverse reactions to the study drugs, cardiovascular, respiratory, hepatic, renal, endocrine, or neurological disorders, autonomic dysfunction with a baseline heart rate (HR) less than 60 beats per minute, hypotension with a systolic blood pressure (SBP) less than 100 mmHg at baseline, current use of medications that could affect autonomic orhemodynamic responses (such as β-blockers, calcium channel blockers, angiotensin-converting enzyme (ACE) inhibitors, α₂-agonists, or antidepressants), bleeding or coagulation abnormalities, infection at the injection site, or spinal deformities.

Sample size estimation

The primary outcome of this study was the duration of the sensory block (in minutes). The necessary sample size was calculated for a two-arm, parallel-group design that compares independent means, with a significance level (α) of 0.05 and a statistical power (1-β) of 95%. Effect size estimation was based on data reported by Saha et al., who compared 5 µg and 10 µg intrathecal dexmedetomidine [8]. The average sensory block duration was 235.6 ± 28.4 minutes for the 5 µg group and 296.2 ± 31.5 minutes for the 10 µg group in their study [8]. The standardized mean difference (Cohen's d) was computed using these values as d = (296.2 - 235.6)/pooled SD, resulting in d = 60.6/30.3 ≈ 2.0. To avoid overestimation due to single-study variability, a conservative effect size of 1.0 was used for power calculations. The sample size was calculated using the formula for two independent means: n = 2 × (Z₁₋⍺/₂ + Z₁₋ᵦ)²/d². In this context, Z₁₋⍺/₂ is equal to 1.96, and Z₁₋ᵦ is equal to 1.645, which corresponds to a power of 95%. Substituting the values: n = 2 × (1.96 + 1.645)²/(1.0)² = 26.00. Thus, the required sample size was approximately 26 participants per group (52 in total). To account for potential attrition or protocol deviations (estimated at 15-20%), the sample size was pragmatically increased to 30 participants per group, resulting in a final total of 60 participants.

Randomization, allocation concealment, and blinding 

Participants were randomized into two groups using a computer-generated simple randomization sequence created by an independent statistician not otherwise involved in study conduct. Allocation concealment was ensured using sequentially numbered, opaque, sealed envelopes (SNOSE). A research coordinator who was not part of data collection enrolled the participants, while a separate anesthesia technician, also not involved in outcome assessment, opened the envelopes and prepared the study syringes according to group allocation.

This trial was conducted using a double-blind design. Participants, the attending anesthesiologist who administered SA, the intraoperative observer, and the data analysts were all blinded to group allocation.

Blinding was maintained by having an independent anesthesiologist prepare the study drugs in identical 3 mL syringes labeled only with the patient’s study code. Both preparations (5 µg and 10 µg dexmedetomidine with 0.5% hyperbaric levobupivacaine) were colorless and indistinguishable in volume and appearance.

The anesthesiologist administering the spinal block and the postoperative assessors had no access to the allocation list or drug preparation area, ensuring the integrity of blinding throughout the study.

Intervention

The doses of 5 µg and 10 µg intrathecal dexmedetomidine were selected based on previous literature indicating that doses within this range enhance sensory and motor blockade without neurological complications. Doses above 10 µg have been linked to significant bradycardia and hypotension [8-11]. Therefore, five µg was chosen as a lower, safer dose, while 10 µg was used to assess the upper limit of efficacy, enabling a direct comparison of the efficacy-safety balance. The enrolled patients were randomly assigned to two groups: Group D5 and Group D10. Patients in Group D5 received 3.0 ml of 0.5% hyperbaric levobupivacaine combined with 5.0 µg dexmedetomidine as an adjuvant. In contrast, those in Group D10 received the same volume and concentration of hyperbaric levobupivacaine, along with a higher dose of dexmedetomidine (10.0 µg).

Preparation of study drug

For Group D5, 5 µg of dexmedetomidine was diluted with normal saline to achieve a final volume of 0.2 ml, which was added to 2.8 ml of 0.5% hyperbaric levobupivacaine to yield a total of 3.0 ml. For Group D10, 10 µg of dexmedetomidine (0.2 ml) was added directly to 2.8 ml of hyperbaric levobupivacaine. All syringes were prepared under sterile conditions using aseptic technique. No visible differences existed between the syringe contents.

Anesthetic management

Before administering SA, the standard monitoring recommended by the ASA, including non-invasive blood pressure, pulse oximetry, and electrocardiogram, was done. After securing an IV cannula, the patient was started on crystalloid solutions and maintained as per requirement intraoperatively. Under all aseptic precautions, a subarachnoid block was administered at the L3-4 or L4-5 intervertebral space using a preloaded drug (blinded) after verifying unobstructed cerebrospinal fluid flow.

Assessment of the block

Evaluation of the onset of sensory block was performed using a cotton swab dipped in alcohol skin prep solution, with loss of temperature sensation noted every three minutes along the mid-clavicular lines until the same dermatomal level was observed on four successive assessments. Motor blockade was assessed using the 4-grade modified Bromage scale, in which 0 indicates full flexion of the knees and feet, and 3 indicates complete inability to move the knees and feet [11]. Along the midclavicular line, intraoperative analgesia was assessed at 15 and 30 minutes, then every 30 minutes thereafter until the block reached the desired T8 dermatomal level. Additionally, non-invasive hemodynamic indices, including HR, SBP, diastolic blood pressure (DBP), and mean arterial pressure (MAP), were recorded at baseline, at induction, and subsequently at 5-minute intervals for the initial 15 minutes after SA.

Outcome measures

The primary outcome was the assessment of sensory blockade to ensure adequate intraoperative analgesia at the target dermatome level T8. Secondary outcomes included evaluation of motor blockade using the modified Bromage scale and monitoring of non-invasive hemodynamic parameters, specifically HR, pulse rate (PR), SBP, DBP, and MAP, to compare physiological stability between the study groups.

Standardized definitions of adverse events

Hemodynamic complications were defined a priori. Hypotension is defined as a ≥20% reduction in SBP from baseline or SBP < 90 mmHg, managed with fluid bolus and ephedrine as required. Bradycardia is defined as HR < 50 beats/min and treated with intravenous atropine 0.6 mg. Desaturation is defined as SpO₂ < 94% on room air. All adverse events and unintended effects were recorded for each group in accordance with the Consolidated Standards of Reporting Trials (CONSORT) 2025 harm-reporting extension.

Statistical analysis

Data were analyzed using Statistical Package for the Social Sciences (SPSS) software, version 26.0 (IBM Corp., USA) [12]. Continuous variables, such as age, hemodynamic parameters (HR, SBP, DBP, MAP), and block characteristics (e.g., time to maximum motor/sensory block), were analyzed using unpaired t-tests to assess mean differences between the two groups. Categorical variables such as gender distribution were compared using Fisher’s Exact Test rather than the Chi-square test because of the small sample size (n = 30 per group) and low expected cell frequencies (< 5) in some categories. Fisher’s Exact Test provides a more accurate estimate of statistical significance under these conditions. The modified Bromage scale, an ordinal measure of motor block, was evaluated using the non-parametric Mann-Whitney U test, as the data were presented as medians with interquartile ranges (IQRs). All tests were two-tailed, and P values < 0.05 were considered statistically significant.

## Results

**Figure 1:**
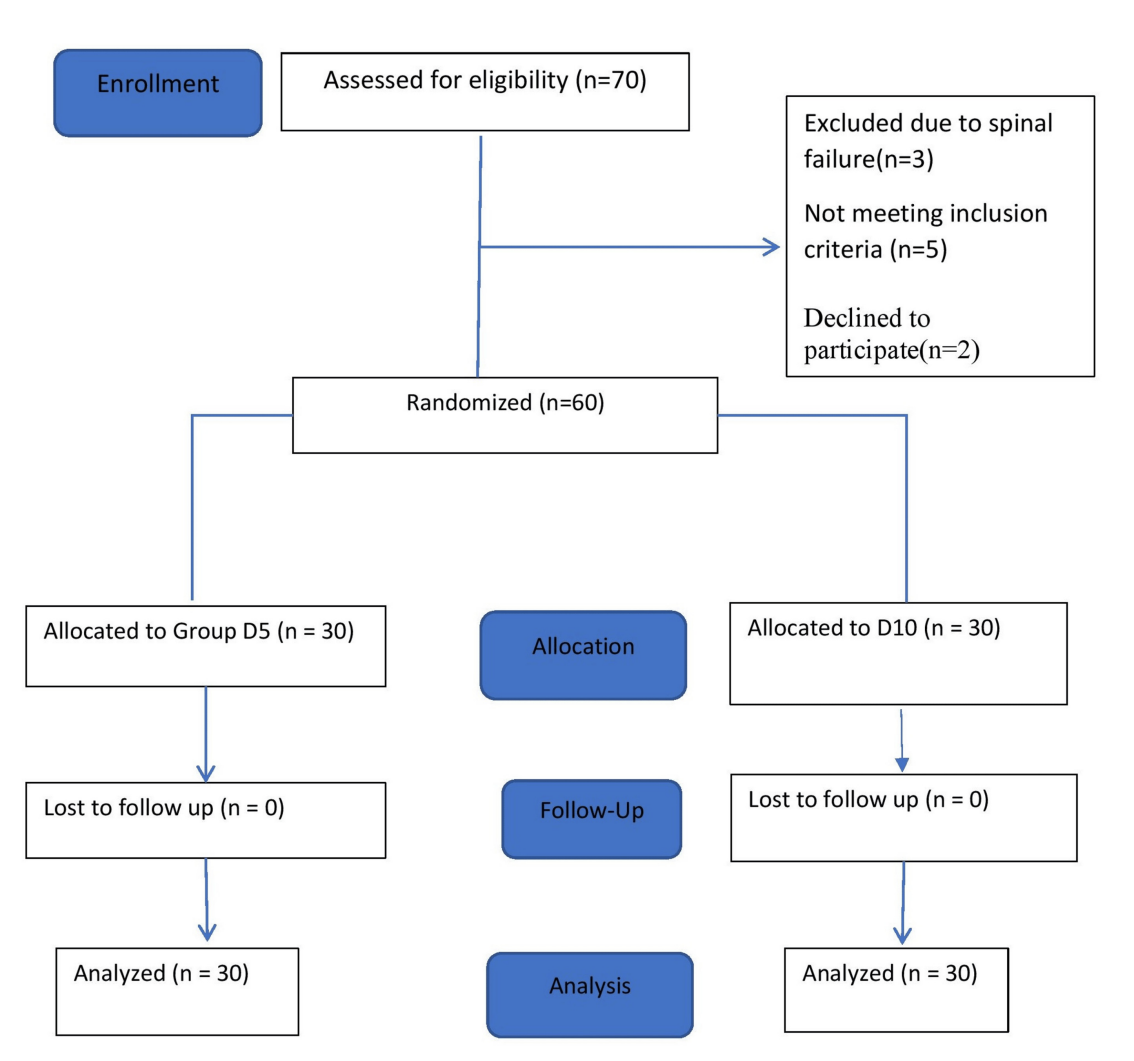
CONSORT flow diagram for enrolment, group allocation, follow-up, and analysis CONSORT: Consolidated Standards of Reporting Trials

A total of 70 patients scheduled for lower-limb surgery under SA were assessed for eligibility. Five subjects were excluded for not meeting the inclusion criteria; three were excluded due to spinal failure, and two declined to participate. Thereby, 60 subjects were randomized equally into two groups: Group D5 (n = 30) and Group D10 (n = 30). There were no losses to follow-up in either group (Figure 1).

The mean age, gender distribution, SBP, DBP, MAP, and HR were similar between the two groups (Table 1).

**Table 1:** Baseline demographic and clinical characteristics of participants Statistical tests: Independent-samples t-test for continuous variables; Chi-square test for categorical variables. SBP: Systolic blood pressure; DBP: Diastolic blood pressure; MAP: Mean arterial pressure; HR: Heart rate P < 0.05 is considered statistically significant.

Variable	Group D5 (n = 30) Mean ± SD / n (%)	Group D10 (n = 30) Mean ± SD / n (%)	Test Statistic (df)	p-Value
Age (years)	38.03 ± 13.94	33.83 ± 10.31	t(58) = 1.33	0.19
Male (n (%))	18 (60.0)	17 (56.7)	χ² (1) = 0.07	0.79
Female (n (%))	12 (40.0)	13 (43.3)	-	-
SBP (mmHg)	127.03 ± 12.65	127.57 ± 12.24	t(58) = 0.17	0.87
DBP (mmHg)	77.60 ± 11.06	77.60 ± 11.06	t(58) = 0.00	> 0.99
MAP (mmHg)	93.73 ± 8.59	93.93 ± 8.69	t(58) = 0.09	0.93
HR (bpm)	85.03 ± 16.17	90.67 ± 14.09	t(58) = 1.45	0.16

Group D10 achieved the maximum motor and sensory blocks faster (p = 0.0001 and p = 0.0172, respectively) and had longer durations of block recession to T8 and of the sensory block (both p < 0.0001). These results indicate that Group D10 experienced a more rapid onset and prolonged duration of sensory and motor blocks than Group D5, highlighting the impact of the intervention (likely drug dosage) on block efficacy (Table 2).

**Table 2:** Comparison of block characteristics between Group D5 and Group D10 (onset time and duration of blocks) † Time to maximum sensory and motor block was considered equivalent to onset time, i.e., the time interval between intrathecal injection and attainment of the highest sensory level or complete motor blockade (modified Bromage scale = 3). Statistical test: Independent-samples t-test p < 0.05 is considered statistically significant

Parameter	Group D5 (n = 30) Mean ± SD	Group D10 (n = 30) Mean ± SD	t-Value (df = 58)	p-Value
Time to Maximum Motor Block (Min) †	7.90 ± 2.12	6.05 ± 1.30	4.11	0.0001
Time to Maximum Sensory Block (Min) †	4.40 ± 1.22	3.65 ± 1.15	2.44	0.017
Time for Block Recession up to T8 (Min)	70.83 ± 11.82	98.67 ± 11.68	7.42	< 0.0001
Duration of Sensory Block (Min)	223.90 ± 29.83	306.80 ± 28.45	9.99	< 0.0001

Table 3 compares modified Bromage scale scores across groups over time. While both groups initially had identical median scores (3), significant differences emerged after three hours, with Group D10 maintaining higher scores (indicating greater motor block) than Group D5 (p < 0.0001). This suggests that Group D10 sustained motor block effectiveness longer, further supporting the prolonged efficacy observed in Table 2.

**Table 3:** Comparison of modified Bromage scale between Group D5 and Group D10 Data are presented as median (IQR). p < 0.05 was considered statistically significant. Data were analyzed with Mann-Whitney U test. IQR: Interquartile range

Time (post-induction)	Group D5 (n = 30), Median (IQR)	Group D10 (n = 30), Median (IQR)	p-Value (Mann–Whitney U test)
15 min	3 (3–3)	3 (3–3)	> 0.9999
30 min	3 (3–3)	3 (3–3)	> 0.9999
1 hr	3 (3–3)	3 (3–3)	> 0.9999
1.5 hr	3 (3–3)	3 (3–3)	> 0.9999
2 hr	3 (3–3)	3 (3–3)	> 0.9999
2.5 hr	3 (3–3)	3 (3–3)	> 0.9999
3 hr	2 (2–3)	3 (3–3)	< 0.0001
3.5 hr	2 (1–2)	3 (3–3)	< 0.0001
4 hr	1 (1–2)	3 (3–3)	< 0.0001

**Figure 2:**
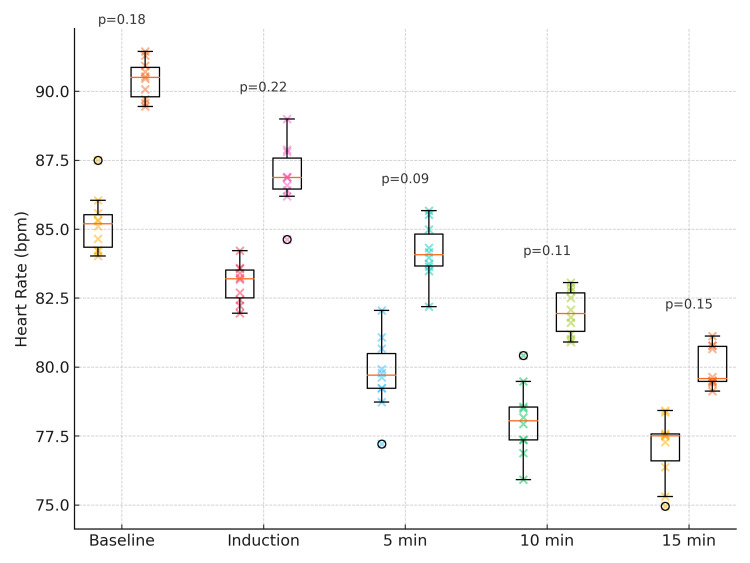
Comparison of HR Between Group D5 and Group D10 Box-and-whisker plots (with jittered individual data points) show the distribution of HR (beats per minute) at baseline, during induction, and at 5, 10, and 15 minutes after SA in Group D5 (5 µg dexmedetomidine) and Group D10 (10 µg dexmedetomidine). Boxes represent IQRs, horizontal lines indicate medians, whiskers show the 10th-90th percentiles, and scattered points depict individual observations. P-values for each time point are shown above the corresponding box pairs (p < 0.05 is considered statistically significant). Statistical analysis was performed using the independent-samples t-test for continuous data. HR: Heart rate; SA: Spinal anesthesia; IQR: Interquartile range

Both groups exhibited a gradual decline in HR over time, with Group D10 consistently having higher rates, although the difference was not statistically significant (Figure 2).

**Figure 3:**
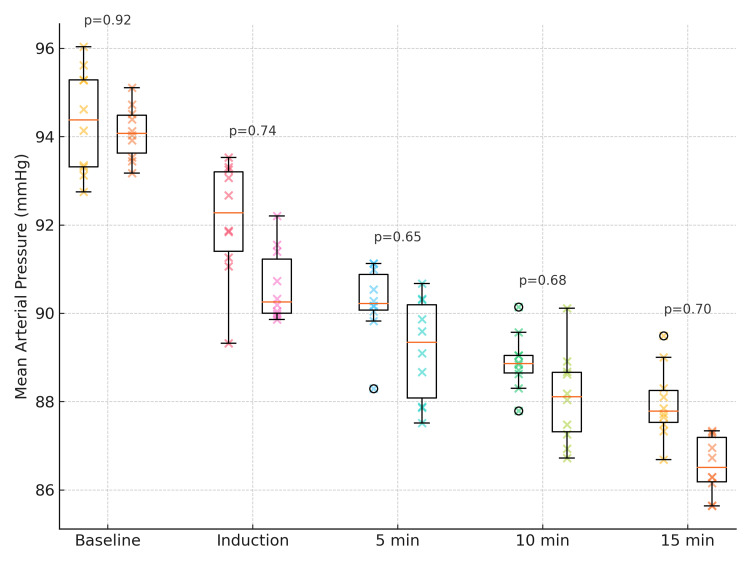
Comparison of MAP between Group D5 and Group D10 Box-and-whisker plots (with jittered individual data points) show MAP (mmHg) at baseline, during induction, and at 5, 10, and 15 minutes following SA in Group D5 (5 µg dexmedetomidine) and Group D10 (10 µg dexmedetomidine). Boxes denote the IQR, whiskers indicate the 10th–90th percentiles, and circles display individual data points to show distribution spread. P-values are annotated above each pair of boxes for direct visual comparison (p < 0.05 is significant). Analysis was conducted using the independent-samples t-test for between-group comparisons at each time point. MAP: Mean arterial pressure; SA: Spinal anesthesia; IQR: Interquartile range

Similarly, SBP, DBP, and MAP decreased progressively in both groups, with nearly identical trends and overlapping standard deviations, indicating comparable hemodynamic stability (Figure 3).

Harms and unintended effects

No neurological deficits, respiratory depression, postoperative nausea/vomiting, urinary retention, or pruritus were observed in either group. In Group D10, bradycardia occurred in five patients (16.7%), and hypotension occurred in six patients (20%); all events were transient and managed successfully with IV atropine or fluid bolus without any need to withdraw participants from the trial. In Group D5, bradycardia occurred in one patient (3.3%), and hypotension in two patients (6.7%).

## Discussion

The present randomized controlled study evaluated the comparative efficacy and safety of two intrathecal doses of dexmedetomidine (5 µg and 10 µg) combined with 0.5% hyperbaric levobupivacaine in patients undergoing elective lower-limb surgeries. The findings confirm a clear dose-dependent enhancement of both sensory and motor block characteristics, along with a predictable increase in hemodynamic side effects at the higher dose.

Block characteristics

Patients in Group D10 exhibited a faster onset and prolonged duration of both sensory and motor blocks compared with Group D5, corroborating previous findings by Saha et al., Halder et al., Al-Mustafa et al., and Naaz et al. [8,9,13,14]. The enhancement of SA by dexmedetomidine is due to α₂-adrenoceptor-mediated suppression of C-fiber neurotransmission, hyperpolarization of dorsal horn interneurons, and promotion of local anesthetic binding at sodium channels.

The mean onset of sensory block (≈3.6 min) and duration (≈307 min) in Group D10 align with earlier studies using similar doses, suggesting enhanced drug synergy at higher intrathecal concentrations [9,14]. Conversely, the 5 µg dose provided sufficient anesthesia for surgeries averaging 90-120 minutes, which reflects the typical duration of orthopedic procedures such as tibial plating or femoral nailing. This supports the clinical feasibility of tailoring the dexmedetomidine dose to surgical duration.

A similar dose-dependent enhancement was observed in motor block parameters. Group D10 showed a significantly shorter onset time and longer duration of motor blockade, consistent with prior studies by Halvadia et al. and Nallam et al. [15,16]. The α2-receptor-mediated inhibition of motor neuron excitability likely explains this effect. Additionally, levobupivacaine’s stereoselective properties may synergize with dexmedetomidine to stabilize neuronal membranes, further prolonging motor blockade without increasing the risk of neurotoxicity. While a more profound and prolonged motor block may be beneficial for intraoperative immobility, it may delay postoperative ambulation, emphasizing the need for dose optimization based on surgical duration and patient recovery goals.

Hemodynamic effects

Although both doses maintained acceptable hemodynamic stability, bradycardia (16.7%) and hypotension (20%) occurred more frequently in the 10 µg group. These effects are consistent with dexmedetomidine’s centrally mediated sympatholysis, which reduces norepinephrine release and enhances vagal tone. The absence of severe or unmanageable events underscores the safety of both doses when appropriate monitoring and timely intervention (with atropine and fluid resuscitation) are implemented.

Importantly, all patients with potential cardiovascular risks or on autonomic-modulating medications were excluded, ensuring that the observed hemodynamic effects were attributable to the study drug. The results therefore provide a reliable comparison of physiological responses in otherwise healthy surgical candidates.

Although both groups maintained acceptable intraoperative hemodynamic stability, Group D10 experienced more pronounced decreases in HR and blood pressure. This observation is consistent with the results of Halder et al. and Naaz et al., who documented markedly elevated occurrences of bradycardia and hypotension at 10 μg doses [9,14]. 

Adverse effects and safety profile

No major neurological or respiratory complications were observed in either group, aligning with the safety profiles reported by Shukla et al. and Owais et al. [17,18]. However, the slightly higher incidence of bradycardia (16.7%) and hypotension (20%) in Group D10 suggests that while 10 μg provides superior block characteristics, it requires heightened vigilance. The absence of neurotoxicity supports the neurocompatibility of dexmedetomidine at this dose range.

Clinical implications

The findings have direct implications for dose optimization in clinical practice. For short to moderately long procedures (≤ 2 hours), 5 µg dexmedetomidine offers a favorable efficacy-safety balance, providing adequate block duration with minimal hemodynamic perturbation. In contrast, 10 µg may be reserved for prolonged surgeries (> 2 hours), where extended anesthesia is desirable and close cardiovascular monitoring is feasible.

Thus, rather than a universal “one-dose-fits-all” approach, dose selection should be individualized based on surgical duration, patient stability, and institutional monitoring capacity. This approach can improve patient comfort, reduce anesthetic supplementation, and minimize postoperative cardiovascular instability.

Limitations and future directions

This study was confined exclusively to lower-limb orthopedic surgeries, which limits generalizability. The results cannot be directly applied to abdominal, cesarean, urological, or non-orthopedic surgeries without validation from subsequent proof-of-concept studies. The study did not assess postoperative or long-term outcomes, such as time to ambulation, postoperative analgesia requirements, or late hemodynamic effects. The lack of long-term follow-up constrains the capacity to derive more extensive clinical conclusions concerning recovery trajectories or enduring safety. Future multicenter trials with larger cohorts and extended follow-up could help refine the optimal intrathecal dexmedetomidine dose across diverse surgical contexts and patient populations.

Several measures were implemented to reduce bias, including computer-generated randomization, allocation concealment using SNOSE, double blinding, standardized anesthetic protocols, and the use of the same investigator for all intraoperative assessments.

Performance bias was minimized by ensuring identical-appearing study syringes. Detection bias was reduced by blinding the outcome assessor. Although observer bias cannot be eliminated entirely in clinical anesthesia research, the methodological safeguards used here strengthen internal validity.

Study strengths 

This study is among the few randomized controlled trials directly comparing two specific intrathecal doses of dexmedetomidine (5 µg vs 10 µg) combined with hyperbaric levobupivacaine in lower-limb surgeries. The double-blind design standardized anesthetic technique, and the comprehensive assessment of sensory, motor, and hemodynamic parameters strengthened the validity of the findings. Clinically, the results provide dose-specific guidance for optimizing SA: 10 µg dexmedetomidine offers superior and prolonged block characteristics suited for lengthy procedures, whereas 5 µg achieves a favorable efficacy-safety balance ideal for shorter or ambulatory surgeries. These insights can help anesthesiologists tailor dexmedetomidine dosing to individual patient profiles and operative requirements, thereby enhancing safety and postoperative recovery outcomes.

## Conclusions

In this randomized, double-blind study, both 5 µg and 10 µg intrathecal dexmedetomidine effectively enhanced the sensory and motor block characteristics of hyperbaric levobupivacaine in lower-limb orthopedic surgeries. The 10 µg dose produced a faster onset and markedly prolonged duration of anesthesia but was associated with a higher incidence of bradycardia and hypotension. In contrast, 5 µg provided adequate block duration with greater hemodynamic stability, offering a more favorable safety-efficacy balance for procedures of typical orthopedic duration.

These findings support a dose-dependent response and underscore the importance of individualized dose selection based on procedural length, patient stability, and monitoring capacity. Nonetheless, since the study population was limited to lower-limb orthopedic surgeries, the findings should not be extrapolated to other surgical categories without additional proof-of-concept studies. Future research with larger, multi-center cohorts and standardized postoperative assessments is needed to refine optimal intrathecal dexmedetomidine dosing across diverse clinical contexts.
